# The Impact on Service Collaboration of Co-location of Early Childhood Services in Tasmanian Child and Family Centres: An Ethnographic Study

**DOI:** 10.5334/ijic.5581

**Published:** 2021-04-29

**Authors:** Kim Jose, Catherine L. Taylor, Rachael Jones, Susan Banks, Joel Stafford, Stephen R. Zubrick, M’Lynda Stubbs, David B. Preen, Alison Venn, Emily Hansen

**Affiliations:** 1 Menzies Institute for Medical Research, University of Tasmania, Hobart TAS 7000 Australia; 2 Telethon Kids Institute and Centre for Child Health Research, The University of Western Australia, Nedlands, Western Australia, Australia; 3 Menzies Institute for Medical Research, The University of Tasmania, Hobart, Tasmania, Australia; 4 School of Social Sciences, University of Tasmania; Private Bag 1340, Launceston, TAS 7250, Australia; 5 School of Social Sciences, University of Tasmania; Private Bag 22, Hobart, TAS 7001, Australia; 6 The University of Western Australia, Subiaco, Western Australia, Australia; 7 Telethon Kids Institute, 5 Hospital Avenue, Nedlands WA 6009, Australia; 8 Telethon Kids Institute and Centre for Child Health Research, The University of Western Australia, 15 Hospital Avenue, Nedlands WA 6009, Australia; 9 Chigwell Child and Family Centre, Department of Education; 4 Bethune St, Chigwell TAS 7001, Australia; 10 School of Population and Global Health, The University of Western Australia, Nedlands WA 6009, Australia; 11 Menzies Institute for Medical Research, University of Tasmania; Medical Science Precinct, Private Bag 23, Hobart TAS 7000 Australia

**Keywords:** early childhood services, place based, ethnography, co-location, collaboration, Australia

## Abstract

**Introduction:**

There is a global trend towards place-based initiatives (PBIs) to break the cycle of disadvantage and promote positive child development. Co-location is a common element of these initiatives and is intended to deliver more coordinated services for families of young children. This paper examines how co-locating early childhood services (ECS) from health and education in Child and Family Centres (CFCs) has impacted collaboration between services.

**Methods:**

This ethnographic study included 130 participant observation sessions in ECS between April 2017 and December 2018 and semi-structured interviews with 45 early childhood service providers and 39 parents/carers with pre-school aged children.

**Results:**

Service providers based in CFCs reported that co-location of services was facilitating local cooperation and collaboration between services. However, insufficient information sharing between services, prioritising client contact over collaborative practice and limited shared professional development remained barriers to collaborative practice. For parents, co-location improved access to services, but they experienced services independently of each other.

**Discussion and Conclusion:**

Co-location of ECS in CFCs contributed to greater cooperation and collaboration between services. However, for the potential of CFCs to be fully realised there remains a need for governance that better integrates service policies, systems and processes that explicitly support collaborative practice.

## Background

The multi-dimensionality of early life disadvantage is well recognised with exposure to adversities such as poverty, developmental vulnerabilities, insecure housing and family violence in childhood shown to impact on outcomes in adult life [1,2]. A collaborative and integrated early childhood service (ECS) system is considered the most effective way to meet the needs of families and children experiencing a range of adversities, [3,4,5,6,7,8]. The Australian state of Tasmania adopted a place-based service delivery model in 2009, co-locating early childhood services (ECS) from health and education in Child and Family Centres (CFCs). Co-locating ECSs in CFCs was intended to deliver more coordinated services for families of young children and support service integration ‘*as opposed to simply moving services to a single site’ [9]*.

The new service delivery model aimed to enhance cross-sectoral collaboration for ECS from health, education and the social sectors to better support families. This cross-sectoral collaboration is known as horizontal integration and is recognised as particularly important for children who may require collaboration between health and education or social services. Health services commonly focus on improving integrating services across the continuum of care (i.e., prevention, community, hospital and tertiary services). This is referred to as vertical integration and while important for addressing issues of care fragmentation [10,11], may be insufficient to meet the needs of families facing multiple adversities. It is recommended that for children and families services should be delivered in convenient community-based locations, as adopted by CFCs, to address access issues [12].

Occurring on a continuum of increasing complexity (i.e., networking, coordination, collaboration, integration) collaborative practice [6,13,14] without full integration is thought to positively impact health outcomes by supporting coordination or strategies to facilitate communication and support [15]. For children and families, the evidence is limited but indicates that enhanced collaboration or integration brings about increased effectiveness in practice that can result in improved outcomes [8,16,17]. Co-location, which in this study is defined as the sharing of physical space and facilities by previously distributed services [18], has been identified as critical for facilitating service collaboration [5,8,19,20,21] and integrated working; alternatives such as ‘virtual teams’ are considered insufficient to build the strong relationships that underpin a shared approach to problem solving [20]. However, co-location of services is not always possible and it is acknowledged that co-location alone does not guarantee collaborative practice [6]. In practice, collaborative service delivery relies on a range of factors including but not limited to; physical proximity, shared goals and resources, joint planning, multi-agency steering or management committees, understanding roles and responsibilities, joint training and support for staff [22].

Previously, we have shown that parents who use CFCs rate their experience of services more highly than those who do not [23] and that CFCs use a range of outreach strategies to engage with families [24]. However, how services in CFCs are working together to support families has been unclear. This paper addresses the question “what is the impact of co-location of early childhood services in Tasmanian CFCs on collaboration between service providers from health and education and integration within the early childhood sector?” The results presented were produced within the Tassie Kids Study which is a partnership between researchers and the Tasmanian Departments of Health, Education, and Premier and Cabinet focused on the investigating the uptake and reach of universal early childhood service system.

## Methods

This study employed ethnographic methods to explore how ECS engage with parents and collaboration between services. An ethnographic study design was selected as it provides in-depth insights into people’s views and actions with respect to their situation or location, through the collection of detailed observations and interviews [25]. Findings are reported according to the standards for reporting qualitative research (SRQR)[26]. Ethical approval was received from the Tasmanian Human Research Ethics Committee (H0016195).

### Sites and services

The study was conducted primarily across two Tasmanian communities (sites); one rural area with a CFC (pseudonym, Distant Hills) and one suburban area without a CFC (pseudonym, Rivertown). Communities were selected based on high socioeconomic disadvantage, number of births and outcomes from the Australian Early Development Census (AEDC) [27] as government partners were interested in engagement with services, particularly among more vulnerable families. To protect participant anonymity, support purposive sampling and in recognition that services located in CFCs may vary in response to the needs of their local community additional data was also collected from two comparable sites within Tasmania with CFCs.

Three key universal early childhood services from health and education operate in Tasmania; Child Health and Parenting Service (CHaPS), Launching into Learning (LiL) and Child and Family Centres (CFC) (see ***Table 1***). CHaPS and LiL are distinct health and education programs available across the state. CFCs are purpose-built buildings operating as a service delivery hub, offering distinct programs and activities for parents and children as well as acting as a site for ECS to operate from. In 2017, 12 CFCs were operating across Tasmania with CHaPS and LiL services operating from CFCs in the communities where they were located. This study focuses on the CFC model of service delivery but as CHaPS and LiL services operate from these locations in some communities and there are plans to expand the CFC model to other communities the findings draw on data collected from all sites [23].

**Table 1:** Summary of Tasmanian Universal Early Childhood Services.


SERVICE	ACRONYM	GOVERNMENT DEPARTMENT RESPONSIBLE	SERVICE CHARACTERISTICS

Child Health and Parenting Service	CHaPS	Department of Health	Eligibility: Children aged 0–5 years Community based: various settings including stand-alone clinics and CFCs. Delivered by Child Health Nurses Screening health and developmental checks for children: 2, 4 & 8 weeks 6 & 12 months 2 & 4 years Appointment based system One open ‘drop in’ session per week Additional targeted services available

Launching into Learning	LiL	Department of Education	Eligibility: Children from 0–4 years School based Delivered by early childhood teachers and teachers aides Support child development, parent/child relationships and facilitate the transition to school Play-based activities, excursions Parents/carers present with children Structured 2-hour sessions Available during school Terms Number of sessions determined by each school. For schools in this study the number of sessions ranged from seven per week to once per week.

Child and Family Centres	CFC or Centre	Department of Education	Eligibility: Children from 0–5 years Twelve sites in Tasmania All Centres staffed by Centre leader, Community Inclusion Worker, Early childhood teacher. Other staff vary across Centres in response to community need e.g., Aboriginal Early Years Support Workers or speech therapists. Health, education and community services offered at the Centre. Operate 5 days per week Open year round Centres offer parent/child groups and activities throughout the week e.g. parenting course, play based activities. Outreach activities include transport, home visits, attending services alongside families. Parents and children can ‘drop in’ at any time


### Data Collection

Data were collected using participant observation sessions and semi-structured interviews with service provider staff and parents. Data collection was undertaken by three experienced female qualitative researchers KJ, RJ and SB between April 2017 and December 2018. At all locations service providers and families were aware of the researcher’s identity and purpose. Researchers did not attend any private consultations between service providers and families.

### Participant Observation

Participant observation (PO) is an open-ended inductive style of data collection where researchers spend time among the group of people or settings they are studying. Researchers observe, participate and talk with participants in informal interviews in order to learn about this particular social world and build an in-depth understanding of practices and culture. Over 130 participant observation sessions were undertaken in health and education ECS settings across the primary sites and two additional sites. Some sessions lasted entire days, others a few hours. Researchers conducted PO in common areas in CFCs, CHaPs clinics, new parent groups, community outreach events and excursions. They also attended meetings held in CFCs, local early childhood network meetings and LiL programs offered by primary schools in Distant Hills and Rivertown.

Researchers used field notes to record observations, informal interviews, conversations and reflections on the experiences of the researchers [28]. A fieldwork activity log was kept by researchers to track their engagement in the field. The extensive period of participant observation facilitated the development of rapport with ECS providers and parents and allowed for researchers to develop a greater understanding of how ECSs operate and engage with families and each other. It also enabled researchers to focus their observations on key elements as they emerged.

### Semi-Structured Interviews

#### Service Providers

Semi-structured interviews were conducted with service providers from CHaPS, LiL and CFCs situated in Distant Hills and River Town as well as the additional sites. Forty-five service personnel were recruited: 17 from Distant Hills, 13 from River Town and 15 from two other sites. Interviews focused on the role of the service, the role of the interviewee within the service, engagement with families including outreach, collaboration with other early childhood services and barriers and facilitators to collaboration, including the impact of co-location on service collaboration. Interviews were conducted on site at the CFCs, schools or clinics and averaged 47 minutes. Service managers gave approval for service providers to participate in this study, participation was voluntary.

#### Parents

Parents with at least one child aged under 5 years were recruited from the key sites and from the additional sites. An effort was made to seek out parents with varying experiences and service use pathways. Researchers approached parents or carers during attendance at an ECS activity, such as the CFC or LiL session to recruit for interviews. Recruitment was also aided by LiL teachers and CFC staff who discussed the study with families or assisted with identifying families for inclusion. Participants were given a choice of interview location. All initial interviews took place at a CFC or the local schools. Some follow-up interviews were conducted in the participants home.

Thirty-nine parents were recruited into the study. One parent was recruited but not formally interviewed as they did not attend the scheduled interview. One couple was interviewed together while the remaining were interviewed alone. We invited all interviewees to participate in a follow-up interview to reflect on experiences recounted in initial interviews as well as capture how service use had changed as children had grown. Of the 38 parents interviewed, 23 parents were interviewed twice over a 12-month period.

Parents received a voucher to the value of $50 for each interview in recognition of their time. Interviews with parents focused on parenting experiences, use and experience of ECS and avenues for accessing parenting support when needed. Parent interviews averaged 33 minutes.

### Data Analysis

Interview audio-recordings were fully transcribed by a transcription service and any relevant interview field notes were attached to the de-identified transcripts before importing into the qualitative data analysis software program NVivo 11 (QSR International 2012). All fieldnotes from the participant observation were also imported into NVivo. Interview transcripts and field-notes were then read and re read several times. They were then analysed thematically using an iterative process that utilized coding and the constant comparison technique [29]. Interview transcripts underwent an initial preliminary analysis soon after the interview was conducted so the researchers could take insights from that interview into any subsequent interviews. Initial codes were developed from the data (a type of open coding) and included many in-vivo codes. Some in-vivo codes were derived from fieldnotes. Following a process of compare and contrast the codes were then sorted, refined and regrouped into higher order conceptual categories. Coding decisions, key concepts ideas and reflections were identified and recorded in the project log and memos [30]. For the purpose of investigator triangulation and to encourage reflexivity the ethnographic research team (KJ, RJ, EH, SB) met regularly to review project memos, compare coding and refine the analysis [31]. Any disagreements were resolved via discussion. For the analysis presented in this paper first author KJ regrouped the codes and initial themes into larger thematic categories relevant to co-location, collaboration and integration. These were reviewed by the group and finalised. Coding decisions, key concepts, ideas and reflections were identified and recorded in the project log and memos [32].

#### Results

Study participants included 39 parents with a child under the age of 5 years and 45 ECS providers from CHaPs, LiL and CFCs (***Table 2***). This included 11 child health nurses/nurse managers (CHaPS) of whom 5 worked in CFCs some of the time, 2 allied health providers (CHaPS, CFCs), 16 early childhood teachers/senior teachers (CFC, LiL) of whom 3 worked from CFCs some of the time, and 16 CFC staff (i.e., CFC Leaders, Community Inclusion Workers, Education Officer, Centre Assistants and Aboriginal Early Years Support Workers). Seventeen service providers located in the primary CFC site, 13 from the non-CFC primary site and 15 from the two additional CFC sites participated. Most CFC observations occurred in the primary site with 12 days of observations occurring in the additional sites. The findings presented here draw on the formal and informal interview data as well as PO fieldnotes and are presented according to five key themes:1) seeking greater collaboration, 2) co-location and collaborative practice 3) barriers to collaboration 4) facilitating collaborative practice and 5) how families’ experience services. Quotes are presented with a participant number and indication of their role. For example, CHaPS nurse (N2), LiL staff (LiL5), CFC staff member (CFC3) and for nurses or LiL service providers whether they were based in a CFC or not (CFC-based, or non-CFC based).

**Table 2:** Participant characteristics.


PARENTS, N	39

**Gender (males = 8, females = 31)**	

Female (%)	80

**Age (average, range) years**	32.9 (18–56)

**Parent age first child (years)**	

First child < 20	12

First child 21–35	23

First child > 35	4

**Number of Children at First Interview**	

One child	7

Two/three children	25

More than three children	16

Average number children	2.6

**Number of services used (self-report)**	

Multiple (including CFC)	24

CHAPS only	2

LiL only	7

Lil and CHAPS	3

Other only	1

**Family Structure First Interview**	

Single parents	10

Partner	29

**Education**	

Year 10 or less	20

Year 11/12 (includes one year 13)	13

Certificate	3

Batchelor	2

Missing	1

**SERVICE PROVIDERS, N**	**45**

**Gender**	

Female (%)	100

**Age, years**	

Average, range	48 (28–74)

**Educational attainment**	

Certificate or below	9

Batchelor	36

**Early Childhood Service**	

CHaPS Nurses	11

Other Health	2

CFC staff*	17

LiL Staff	15

**Timing working early childhood sector**	

Average, range years	13.1 (0.3–54)


* CFC staff have health, education or community sector qualifications or experience.

### Seeking greater collaboration

This theme draws on data collected from all service provider interviews. Many service providers stated that greater collaboration between services would enable them to better meet the needs of families in their community, prevent duplication of services and over-servicing of families, reduce the burden on families, facilitate identification of shared goals and priorities as well as enable a more consistent approach to working with families.

Service providers from health and education such as LiL teachers or CHaPs nurses indicated that they were seeking better coordination and collaboration with other services within their own sector (vertical integration).

What’s not really working particularly well is communication with GPs and us. I think that’s an area that really, really needs to be improved upon. (N4, CFC-based)

I’ve never had a visit from my principal in all the years that I have done Launching into Learning. (LiL4, non-CFC based)

Many CFC staff, in contrast, discussed a desire for enhanced collaboration across the health and education sectors (horizontal integration).

It would be really nice if the Department of Health and Human Services could work with the Department of Education, like if there could actually be a cross over between us and them. (CFC11)

Service providers from all government services also reflected on the challenges of working collaboratively with the non-government sector and other government programs such as income or employment services whose work impacted on families.

### Co-location and collaborative practice

This theme draws on interviews with all service providers and observations undertaken in CFCs. Service providers from education and health identified co-location of services at CFCs as a mechanism for supporting service collaboration and integration. Service providers from River Town, where there was no CFC, frequently identified the Centre-based CFC model of service delivery when asked what an integrated or collaborative service would look like. They had a perception that ‘*a one stop shop*’ would address potential barriers to accessing services for clients.

Service providers who worked in CFCs affirmed that co-location does facilitate connection with services for families, particularly discussing the benefits of ‘*warm referrals’ for* parents. ‘Warm referrals’ are personalised referral processes that support families to engage with other services. Warm referrals, while informal in nature, were a deliberate strategy to support family’s engagement with ECS that was supported by having services co-located as this LiL teacher revealed.

our health nurse – and this is the most amazing thing about this whole place – would have a warm referral, talk to parents, “I would love you to meet [CFC staff member] because she can work with people in really small groups and things. How would you feel about that?” So on the spot out we go and we meet this family and set up a time to catch up and so that would be the way and they would be sustainable. (LiL6, CFC -based)

Whereas a nurse who worked in multiple locations noted that ‘*somewhere else it would be an actual referral, so that [co-location] makes it easier*’. (N4, CFC-based).

CFC workers reported that due to co-location of services in CFCs families have greater knowledge of the services available to them and their purpose, had experienced improved access to services supported by informal connections that built trust, and that the service setting had increased the capacity for services to work together with families and develop shared goals.

In addition to informal collaborative practices CFCs enabled staff to undertake targeted collaborative activities. Where the CFC was situated next to the local primary school this collaboration extended to include school-based staff (see Additional file 1). While physical co-location or proximity was identified as facilitating collaborative practice some service providers recognised that collaboration was not solely reliant on co-location as this CFC based nurse outlined.

I’ve realised it’s not about buildings. It’s about just that collaborative practice and being within a physical proximity to each other so that you can see it. Because they’re not going to get buildings like this everywhere. It’s not the building. (N11, CFC-based)

While stating that collaboration is not about the building this nurse did recognise the value of being co-located, supporting co-location of a clinic in another town. This was now acting as a ‘*hub and the families are loving it, because they get that connection’ (N11, CFC-based)*.

### CFC strategies facilitating collaborative practice

A range of collaborative practices and activities were observed by researchers in CFCs and discussed by service providers working from CFCs. This included child health nurses and CFC staff making home visits together. These combined home visits were undertaken in order; “*just to connect them with the Centre”. (N1, CFC-based)* so that “*when they do come in, they know a familiar face*” (CFC15). In one Centre ‘open, drop in’ sessions offered by the child health nurse had been scheduled to coincide with the CFCs ‘baby’s group’ and staff at the local primary school had reviewed their roster to ensure they had flexibility to spend time at the CFC (see Additional file 1). In one CFC all new parents on their first visit to the CHaPS nurse were introduced to CFC staff;

*We (CHaPS nurse and CFC worker) came up with the idea that I would ask mothers at the eight week check, if they were happy for [CFC staff member] to spend 10 minutes with them (N11, CFC-based)*.

At one site the CHaPS nurses led one session of the six-week parenting course. Only one of the three CFCs in this study used all of these practices (i.e, joint home visits, rostering/scheduling and systematic introduction to CFC staff).

Two of the CFCs coordinated regular meetings for LiL and kindergarten teachers and support staff from primary schools in the area for networking and professional development opportunities. In one site the explicit aim of the group was to ensure no child arrived at school without having prior contact with one of the local early years’ services.

CFC governance mechanisms were designed to support collaborative practice with all CFCs having an advisory body whose purpose is to facilitate community input into service design. These groups comprise community members and representatives from early childhood services available in CFCs (e.g., health, education, childcare) and were observed to meet with varying frequency and formality across the Centres. Meetings provided opportunities to update members on what had been happening in the Centre, seek feedback or input into future activities and identify any key changes within the Department of Education. These groups, while acknowledged as valuable for facilitating community input into Centres, had no formal input into governance of CFCs.

Each CFC have developed Working Together Agreements (WTA) that incorporate Family Partnership principles (i.e., build parents capacity to use their own resources and manage problems, engage parents and develop relationship with them that is supportive in and of itself, understand families in a holistic way, work in partnership with families; relinquishing the role of expert to work with rather than lead families) [33]. The WTA use plain language to articulate a shared service delivery framework and family-centred model of care [9] but have largely focused on how service providers work with families rather than how services work together. For example, keep children at the centre of every decision, always be welcoming, keep information private, be encouraging and nurturing, accept that everyone has different ways of doing and seeing things, set a good example for our children.

Observations and conversations with service providers revealed that collaborative practice was also influenced by individual practitioners’ commitment to working in this way, frequently in the absence of governance structures.

… what we need moving into the future is making sure that we really have got those systems in place. It doesn’t matter who’s in each of our roles, that we’ve got the policy behind to support the way we work. (CFC13)

The range of approaches adopted to support collaboration in CFCs were acknowledged by most CFC service providers as contributing to collaborative practices, but they considered collaboration was not sufficiently embedded within CFC service systems and structures

### Barriers to collaboration between service providers co-located in CFCs

Drawing on observations and interviews with service providers working from CFCs three key barriers to collaboration in CFCs were identified. These are governance restrictions around information sharing, prioritising client contact over non-client based activities such as attending cross-agency meetings and lack of a shared understanding of professional practice.

#### Information Sharing

The most significant barrier to more collaborative practices identified by practitioners was governance restrictions on sharing service records and information about children and families between health and education services. The complexities around managing the restrictions on information sharing were considered by some service providers to be an impediment to services meeting the needs of children and parents:

It’s the sharing of information that’s definitely the greatest challenge. … for the actual people in the building, it is the sharing of information and being able to have time … to sit and have a talk. (CFC7)

For example, the CHaPS service receive notification and contact details about all new parents following discharge from hospital while CFC staff, who are employees of the Department of Education, do not receive this information. CFC staff frequently indicated that they were seeking similar notification so that they could engage with all new parents in their area:

I just want them to give me the births at the [local] Hospital, that’s all I need. … but we don’t get any of that information. … education policy is … from birth. The learning of children from birth, but we still can’t get it. (CFC5)

Service providers discussed the potential benefits sharing information such as birth notifications and contact details for new parents, service use, case summaries, child development and child safety alerts between services. However, this type of sharing was rare due to the legal and policy requirements for protecting client privacy. CHaPS nurses were particularly worried about the legality of sharing information due to the legislation that governs health service providers; ‘*There are rules about what you can and can’t share*’ (N6, non-CFC based). They found the proximity of services in CFCs and expectations from some other service providers such as CFC staff that they would share information to be a source of tension:

Confidentiality, how do we deal with this? Because the nurse needs to be so confidential. It’s so tight and you come to space like this [CFC] … it’s just different. (N11 CFC-based)

A range of strategies that did not compromise confidentiality requirements were adopted by providers to facilitate information sharing for the purpose of collaboration. This included CHaPS nurses introducing parents to CFC staff following appointments, CFC staff introducing themselves to parents while they waited for appointments, directly seeking permission from parents to share contact details and, in some circumstances, conducting joint home visits with families, with permission from families. Where parents indicated that they did not want their contact details shared this was respected.

The negative consequences of not sharing information were raised in interviews by service providers from both health and education and included over-servicing of families, duplication of services, families having to repeat their stories, lack of consistency in approach and impeding shared goal and priority setting with families. Numerous examples were provided of the consequences of not being able to share information between services such as that outlined below.

One day I put in all [these supports] in place for this woman, and then found out, an hour later, that they [CFC workers] were doing exactly the same things for the same woman. (N3, CFC-based)

Service providers suggested various strategies that would enable them to mitigate the current challenges relating to information sharing. These included seeking consent from families accessing services in CFCs to share information between all or selected services, completing a memorandum of understanding between the Departments of Health and Education that would address processes for information sharing in CFCs, and discussing information sharing more openly with families who use the CFC so that they were clear about what information might be shared, with whom and why.

I think if a lot more families knew … then they’d [families] be much more inclined to say, “Oh actually maybe that sounds okay”. … I think the perception … why the information gets shared and what we’re hoping to achieve can be quite a challenge (CFC10)

Participants indicated that agreement between agencies for a state-wide approach to information had not yet been reached.

#### Prioritising client contact

In addition to the challenges associated with managing information sharing, service providers were prioritising client contact over collaborative practices such as cross-agency meetings. A ‘*lack of time*’ was commonly used to delineate this barrier to collaboration. Collaborative practices, such as attendance at cross-agency meetings, were recognised as valuable but also ‘*time consuming*’ and were given a lower priority than direct client contact.

*I’d love to attend [CFC meetings] but every time I’ve put an appointment in or to go, something’s come up, or I’ve got really busy or I need to see a newborn (N1, CFC-based)*.

There was an implication that collaborative activities could only be undertaken if other work demands permitted. In some circumstances service managers from CHaPS, who were not located in the area, attended collaborative meetings in lieu of the practitioners so as not to impact on their case management. This was particularly the case for child health nurses and LiL teachers whose work settings and practice were more structured and less flexible compared with CFC based workers.

I’d really like some extra time to go and see [service providers] … and talk to them more, go in there, but the time is the issue and it’s not seen as on task when you’re in a school situation. I think that’s the tricky part of it. (LiL8, non-CFC based)

#### Developing shared understanding of professional practice

This theme draws from interviews will all service providers pertaining to Family Partnership training, professional boundaries, expectations of services and observations in CFCs. Service providers raised concerns about unrealistic expectations of services, lack of knowledge of each other’s service and skills as well as concerns about maintaining professional boundaries.

Look the only knowledge I have of the CHaPS nurse is what I went through as a parent. … So I suppose I’m not seeing their perspective. They’re probably not really seeing our perspective either. (LiL3, non-CFC based)

LiL teachers valued the opportunity provided by the CFC to connect them with other services in their area and learn about their roles.

Before as LiL [teachers] you’d go to professional development, and nothing was really relevant to your end of the school. … we needed to know all those things that they’re [CFCs] providing: … services for all these young children and that was always really hard to access. … So [the CFC]’s just made a huge difference. (LiL1, non-CFC based)

Except for Family Partnership Training, there were no observed opportunities for inter-professional learning between health and education early years’ service providers. In contrast with some nurses who indicated that family partnership training ensured a common framework for engaging with families, one CHaPS nurse reported that they thought Family Partnership Training would have been more useful if they had attended only with other nurses.

I found it [family partnership training] actually quite a frustrating process … I think it would have been – it would have been nice for us as a whole service to do it all at once. (N4, CFC-based)

CHaPS nurses held monthly nurse education days and LiL teachers and CFC staff had access to professional learning through the DoE Professional Learning Institute program, although limited opportunities for early childhood specific professional development was identified as a concern by some LiL teachers. The CFC at the primary site was observed to organise some local professional development opportunities as part of its regular meetings with LiL teachers in the area. For example, inviting a speech therapist to speak at one of their meetings.

### How families experience services

This theme drew primarily on observations of how services interacted with families in CFCs supplemented by parent interviews. Parents’ discussions about service provided at the CFCs revealed that CFCs facilitated access to services and addressed some of the barriers to access, such as transport; ‘*they also offer us the transport services here and will bring people into the centre as well who don’t have their own transport’ (Parent1, CFC)*. None of the parents discussed receiving joint home visits from CHaPS nurses and CFC workers but observations were made of these visits being planned. A few parents reported that they experienced interdisciplinary or multi-disciplinary services in practice. This was most commonly referred to by parents whose children accessed therapy services with parents reporting therapists attending other services; ‘*[my child’s] workers [therapists] would go to the childcare to see him over at childcare and see how he’s being in a different environment’ (Parent2, CFC)*. When parents discussed service interactions their comments revealed that they experienced such interactions as discrete rather than integrated. Services may be co-located in CFCs but operated as distinct services.

One of the ladies up here [at the CFC] – told me about it [early intervention service] and they made the appointment just to meet up with one of them and we met up one time here before it started. (Parent3, CFC)

A few families who were accessing specialised services for their children discussed having to choose between the services they used rather than accessing interdisciplinary services.

*Like I said I was coming twice a week [to LiL] but I just don’t have the time for it with the appointments I’ve got for myself and (child) at [specialist services]. (Parent4, non-CFC)*.

## Discussion

This study found that co-location of ECS in CFCs was contributing to cross-sectoral collaboration with CFCs contributing to new structures and approaches (e.g. local advisory group, joint home visits, aligning schedules, warm referrals) that facilitated collaborative practice. Thus, enabling local service providers working together where possible to respond to the needs of families. However, co-location had not addressed all impediments to collaborative practice such as information sharing between services, prioritising client contact over collaborative practice and limited interprofessional learning. These factors have all previously been identified as impacting on collaboration and integration in ECS systems [8,17,19,34,35] highlighting that co-location needs to be supported by additional strategies that enhance collaborative practice [5,6,8,19,20,36]. The following discussion outlines why these factors remain challenging to address in practice.

Having mechanisms for sharing information and data between organisations and agencies is a key component in collaborative or integrated practice [5,37], but appears not to be implemented systematically. Multiple layers of legislation, regulation, codes and policies can result in a lack of clarity around managing privacy and confidentiality resulting in unnecessary risk aversion to data sharing. There was tension for service providers in this study between abiding with privacy and confidentiality requirements and collaborative practice that was perceived to have a negative impact on outcomes for children and families. This was a pressing challenge for those who worked in CFCs where service providers were interacting with families and each other daily. Whether these challenges are greater when attempting to collaborate across sectors (i.e., horizontal integration) rather than within sectors (i.e., vertical integration) is not clear.

Similar to the findings of reviews of collaborative and integrated practice this study found that organisational structures, processes and procedures had the capacity to impede or enable collaborative practice [8,19,34]. Shared planning, development of shared practice frameworks as well as resources and infrastructure that supports the development of skills to work collaboratively are considered essential for facilitating collaborative practice [19,34]. Performance frameworks and mechanisms that specify and reward collaboration are also needed to progress collaborative working [19]. These mechanisms were not operating in the organisations represented in the Tassie Kids study but have potential to embed collaborative practices across the ECS system wherever services are located.

Inter-professional learning has been identified as a factor that can enhance collaboration by generating a greater understanding about the roles and expertise of professionals working in other sectors [8,17,34,38]. To effectively support children and families with complex needs interprofessional learning beyond that of health care workers may be required [39].

Ultimately, collaborative and integrated practice is designed to improve the outcomes for the families and children who use the services. As has been previously shown [23], CFCs are facilitating access to services by families and supporting them to develop new skills such as parenting practices [40]. The ‘*warm referrals*’ or ‘soft entry points’ to services facilitated by co-location of services in CFCs are recognised as important for engaging with parents who may not usually access services [21,23,41,42]. However, families in CFCs currently experience services as distinct and separate rather than transdisciplinary, an approach that has been identified as important for effective intervention where the needs are complex [6,43].

This study has some limitations. Researchers did not have access to all service policies and procedures for review. Parents were not asked about their perspectives on information sharing. Researchers were not present during consultations with families and may have missed collaborative practices undertaken with families or away from key sites. However, the extensive fieldwork in CFCs, consistency of interview data from across the three CFCs along with observations provided important insights into how co-location was impacting collaborative practice.

## Conclusions

The CFC-based service delivery model has facilitated greater collaboration at policy, local and service delivery levels. The ‘warm’ referral processes and collaborative practices enabled by co-location has enhanced engagement between service providers and families. However, for the potential of CFCs to be fully realised at a service delivery level and to facilitate interdisciplinary teamwork there remains a need for governance mechanisms such as policies, systems and processes to continue to evolve to support collaborative practice.

## Additional File 1 Vignette

At one of the additional CFC sites researcher KJ was present one morning when, at the end of a meeting, one of the CFC workers, the CHaPS nurse and senior teacher from the local primary school discussed a family and child they were all concerned about. The child was due to start kindergarten the next year, but the family had not responded to the school’s multiple attempts to contact them. Following discussion agreement was reached that the nurse would conduct a joint home visit with the school staff member in the next couple of weeks. During the subsequent interview with the school staff member involved, they reflected that: *We’re fortunate here, I guess we’ve worked really hard this year at us all being responsible for all families, in our little pocket … so just now working in with [child health nurse], are coming regularly in touch with us … “okay we need to drive to [local town] and go and visit this family”, because they will be a pre-kinder family so it’s looking at flagging the families but putting all our heads together on how best to reach them*. This collaborative way of working relied on having a supportive team and adopting strategies that enabled teaching staff from the primary school to connect with families at the CFC: *We’ve purposely looked [at] my timetable too this year, so I am a little bit more flexible on a Thursday, for example, to be able to come over [to the CFC], where possible and so there are a few days in the week that I do just touch base. And it might just be a quick 20-minute stop over, say hi, chat, so we make time for that. To make that – to be present. And also, we have our regular meetings too, so that’s myself and our Principal at school – CFC leader and staff here. So we all come together as regularly as we can … our approach this year has been about the team. So we’ll pull everybody in. Which helps for everybody to be on the same page for our families* (School staff member).
